# The effects of aging on the renal function of a healthy population in Beijing and an evaluation of a range of estimation equations for glomerular filtration rate

**DOI:** 10.18632/aging.202548

**Published:** 2021-02-26

**Authors:** Xu Lengnan, Chen Aiqun, Sun Ying, Li Chuanbao, Mao Yonghui

**Affiliations:** 1 Department of Nephrology, Beijing Hospital, National Center of Gerontology, Institute of Geriatric Medicine, Chinese Academy of Medical Sciences, Beijing 100730, P.R. China; 2 The MOH Key Laboratory of Geriatrics, Beijing Hospital, National Center of Gerontology, Institute of Geriatric Medicine, Chinese Academy of Medical Sciences, Beijing 100730, P.R. China

**Keywords:** glomerular filtration rate, evaluation equations, kidney aging, elderly, China

## Abstract

We investigated how age affected renal function in healthy subjects in Beijing and compared different estimated glomerular filtration rate (eGFR) equations. Kidney function was evaluated by five equations: Chronic Kidney Disease Epidemiology Collaboration (CKD-EPI); Modification of Diet in Renal Disease Study (MDRD); the Chinese version of the MDRD (MDRDc); Full Age Spectrum (FAS); and Berlin Initiative Study (BIS). A total of 46,708 subjects were enrolled and followed-up for 3 years. All showed an increase in sCr and a reduction in eGFR with increasing age. Over the 3 years, the eGFR and serum creatinine (sCr) remained unchanged in most subjects. Different equations showed good consistency; the intraclass correlation coefficients (ICC) was 0.849 for males, and 0.817 for females. The CKD-EPI equation yielded higher GFR values than the other equations (according to sCr levels). For subjects aged over 70 years, the BIS equation produced the lowest eGFR values. In summary, we observed that the renal function of individuals was relatively stable with increasing age, although different eGFR equations yielded data that varied across different populations of subjects and sCr levels.

## INTRODUCTION

Owing to the notable increase in the elderly population of China, there is a clear need to investigate the effect of age-related changes in human health. The kidney is an important organ that removes metabolites, waste products, and toxins, from the body, while retaining water and other useful substances by reabsorption. Aging is associated with a functional decline in the kidney that can have a significant impact on health. A global study, carried out in 2017, reported that there were 697.5 million patients with chronic kidney disease (CKD), thus representing 9.1% of the global population [1]. Furthermore, data from the same study reported that there were 132.3 million patients with CKD in China [1]. A number of studies, carried out in different countries, have concluded that kidney function does not decline with age in every individual [2, 3]. In the present study, we aimed to investigate age-related changes in kidney function within the Chinese population.

The ability to clinically evaluate renal function in an efficient and non-invasive manner is critical when planning changes to patient medication. However, the assessment of renal function should not simply rely on the measurement of serum creatinine (sCr) levels. We should also consider the potential effects of age, race, and weight, on serum creatinine. Thus, it is also important to evaluate glomerular filtration rate (GFR) using validated equations, particularly in vulnerable groups such as children and the elderly [4]. The first estimated GFR (eGFR) equation was introduced in 1976 and was known as the Cockcroft-Gault (C-G) equation [5]. Since then, many different equations have been proposed. Unfortunately, these equations have all been associated with certain limitations. Researchers have therefore attempted to develop new alternatives that would be applicable to a wider range of patients and disease severities, including biogeographical region, ethnicity, and age. At present, the most commonly used equations are those recommended by the Modification of Diet in Renal Disease Study (MDRD) [6] and the Chronic Kidney Disease Epidemiology Collaboration (CKD-EPI) [7]. The latter was specifically recommended for the estimation of GFR by the Kidney Disease Improving Global Outcomes (KDIGO) guidelines [8]. More recently, the Full Age Spectrum (FAS) [9] and Berlin Initiative Study (BIS) [10] equations were developed; these equations show a better correlation with age than other equations. In the present study, we selected these five main equations for comparative analysis.

It is evident from the existing literature that the equations used to determine eGFR are constantly being updated, although we have yet to identify the accuracy of these eGFR equations for the Chinese population [11, 12]. The existing equations were mostly derived from data acquired from European and American populations; Asian populations have yet to be fully considered. Another problem with the original derivation of these equations is that they only involved small populations of elderly subjects.

The application of an appropriately validated equation to determine eGFR in the clinic is vital. It is very likely that different equations may overestimate or underestimate the true GFR [4]. Such inaccuracies may lead to inappropriate clinical assessments of renal function, thus resulting in inappropriate medical treatment. Therefore, it is very important that we identify a suitable equation to determine eGFR in the Chinese population, particularly in the elderly.

The purpose of this study was to investigate age-related change in the renal function of healthy subjects in Beijing, and to compare the significance of five different eGFR equations for the assessment of renal function in the Chinese population. Previous research has established that eGFR equations rely on two main variables: serum creatinine and age. Therefore, we focused on the use of serum creatinine to investigate the relationship between renal function and age, rather than eGFR.

## RESULTS

### General characteristics

A total of 46,682 subjects were enrolled in this study; 27,232 were male (58.33%). For each subject, the follow-up period was 3 years. The age of the participants ranged from 18 to 100 years with an overall mean of 46.79±15.83 years (47.80±15.75 years for males; 45.39±15.80 years for females). Overall mean sCr was 70.06±14.50 μmol/L. The sCr of male subjects (78.4±11.38 μmol/L) was significantly higher than that of female subjects (58.50±9.49 μmol/L; *P*<0.000). A total of 4,196 elderly subjects over 70 years-of-age were included in this study; 2,997 of these were male (71.43%).

### Age-related changes in serum creatinine

**Figure 1:**
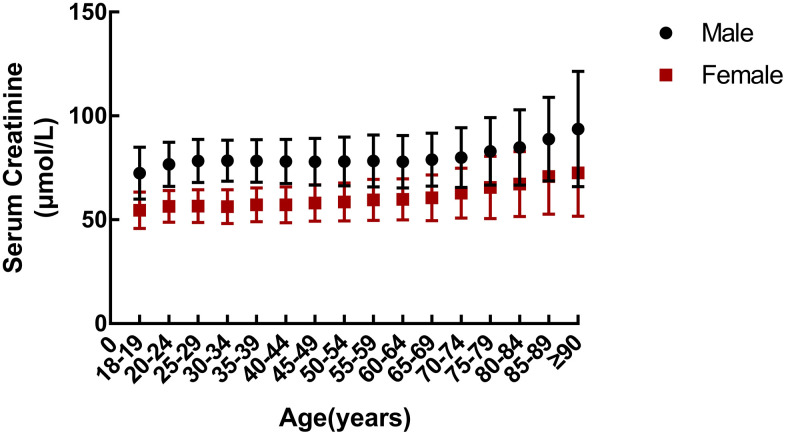
**Age-related changes in serum creatinine in a cohort of healthy subjects.**

Males and females showed similar age-related changes in sCr (Figure 1). Supplementary Tables 1, 2 show sCr levels for all subjects along with statistical comparisons, respectively). SCr increased gradually with age, although the range of sCr change was greater in the elderly population.

### Changes in renal function over a three-year period in different age groups

**Figure 2:**
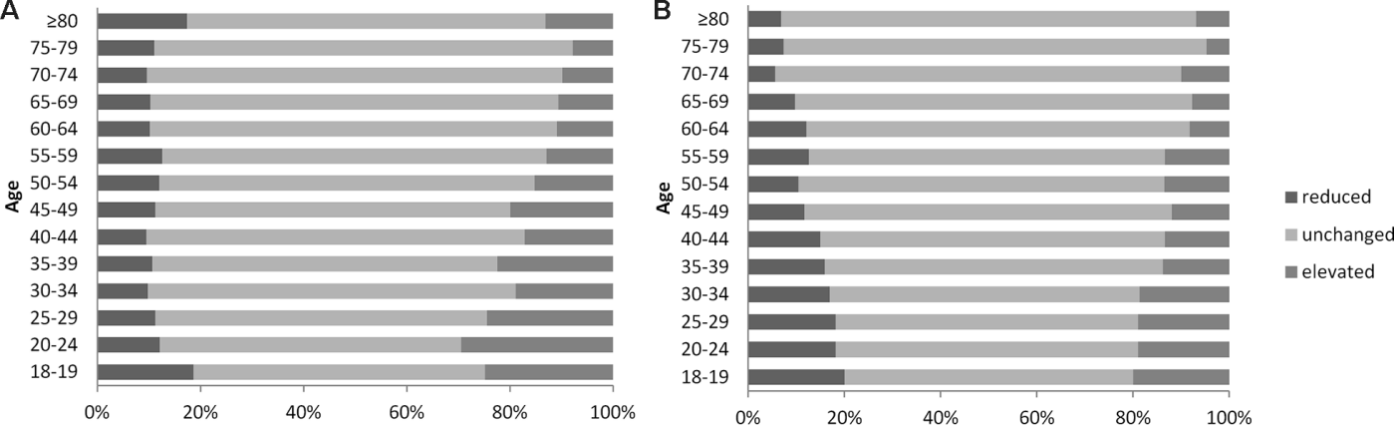
**Changes of estimated glomerular filtration rate (eGFR) in subjects by gender.** (**A**) Male. (**B**) Female. eGFR: estimated glomerular filtration rate.

We used the CKD-EPI equation to calculate changes in eGFR over the three-year study period. For this analysis, subjects were divided into three groups: an ‘elevated’ group (an increase in eGFR that was >5 ml/min/1.73m^2^) [13], a ‘reduced’ group (a decrease in eGFR that was >-5 ml/min/1.73m^2^), and an unchanged group (representing changes that lay between the two other groups). The eGFR of most subjects remained unchanged across the three-year period; thus was more common in the elderly did not show changes (see Figure 2, Supplementary Tables 3, 4). There were still significant differences in the change in eGFR when compared between different age groups (male: χ^2^=248.24, *P*=0.000; female: χ^2^=209.43, *P*=0.000). The eGFR changes and age were negatively correlated (B=-0.27, *P*=0.000). These indicated that the elderly eGFR was less likely to change over a period of time. We also compared changes in sCr across all subjects (Table 1); no significant changes in sCr levels were evident in any of the age groups over the three-year study period.

**Table 1:** Changes of serum creatinine in different age groups of all subjects over a three-year period.

**Age**^#^ **(years)**	**The change of Serum Creatinine (μmol/L)**	
	**Male**	**Female**
<25	0.04±18.58	3.85±13.26
25-29	2.31±15.78	1.84±14.19
30-34	2.69±14.13	1.26±13.48
35-39	2.88±14.12	0.82±12.71
40-44	1.97±14.26	-0.03±12.74
45-49	1.39±14.25	-0.61±13.60
50-54	1.88±14.44	1.20±15.04
55-59	1.04±14.87	2.10±12.84
60-64	1.64±13.44	3.55±18.78
65-69	1.38±12.58	0.76±12.96
70-74	2.47±12.34	3.08±13.74
75-79	1.75±14.51	3.76±13.28
80-84	0.95±15.79	3.98±15.36
85-89	-0.82±17.26	2.93±17.21

^#^For the group ≥ 90 years old, only a few subjects had 3-year follow-up data, so this age group was not analyzed.

### Comparative analysis of different eGFR equations across different age groups

**Figure 3:**
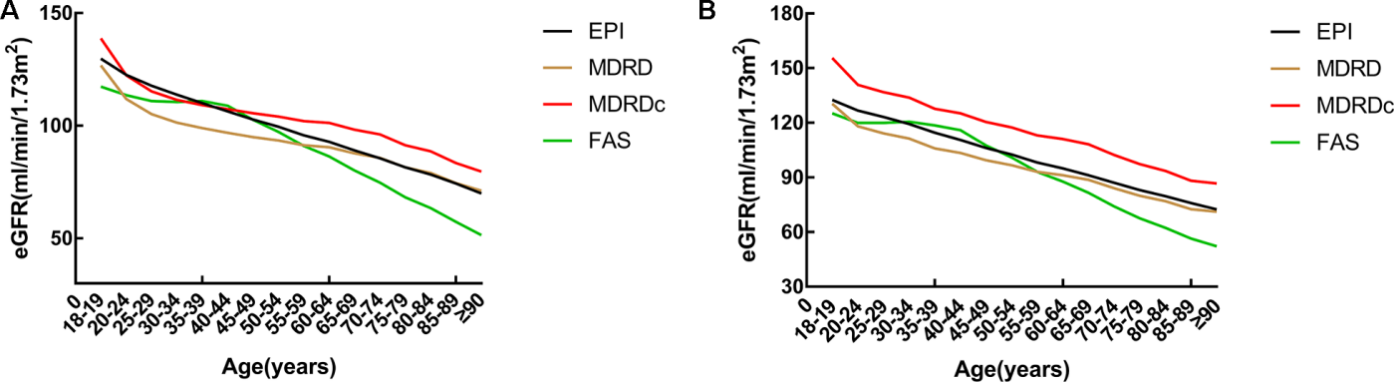
**A comparison of different estimated glomerular filtration rate (eGFR) equations across all age groups.** (**A**) Male. (**B**) Female. eGFR: estimated glomerular filtration rate; CKD-EPI: Chronic Kidney Disease Epidemiology Collaboration equation; MDRD: Modification of Diet in Renal Disease Study equation; MDRDc: Chinese MDRD equation; FAS: Full Age Spectrum equation.

Next, we used the CKD-EPI, MDRD, MDRDc, and FAS equations, to evaluate renal function across the different age groups (Figure 3 and Table 2 and Supplementary Table 5). The highest and lowest values were produced by the MDRDc equation and the MDRD equation, respectively. We also found that there was a significant difference between the four equations (*P*=0.000; Figure 3). Curves created by the CKD-EPI and FAS equations crossed at an age of approximately 40 years. Prior to this crossing point, the FAS equation curve was very flat; after the crossing point, the curve began to decline. In contrast, the CKD-EPI equation curve decreased continuously. Analysis showed that the ICC was very consistent when compared across the different equations; the ICC was 0.849 for males and 0.817 for females.

**Table 2:** A comparison of data produced by the four different estimated glomerular filtration rate (eGFR) equations in all subjects.

	**Male**	t*			**Female**	t*		
		**MDRD**	**MDRDc**	**FAS**		**MDRD**	**MDRDc**	**FAS**
CKD-EPI	101.49±13.02	147.69	-54.12	96.93	106.81±13.88	95.65	-153.52	36.47
MDRD	94.42±17.99		-767.86	-79.42	100.37±21.68		-718.16	-98.65
MDRDc	104.77±20.94			169.73	121.32±27.40			332.24
FAS	97.46±2.02				104.45±24.41			

*P=0.000.

eGFR: estimated glomerular filtration rate; CKD-EPI: Chronic Kidney Disease Epidemiology Collaboration equation; MDRD: Modification of Diet in Renal Disease Study equation; MDRDc: Chinese MDRD equation; FAS: Full Age Spectrum; BIS: Berlin Initiative Study equation.

**Figure 4:**
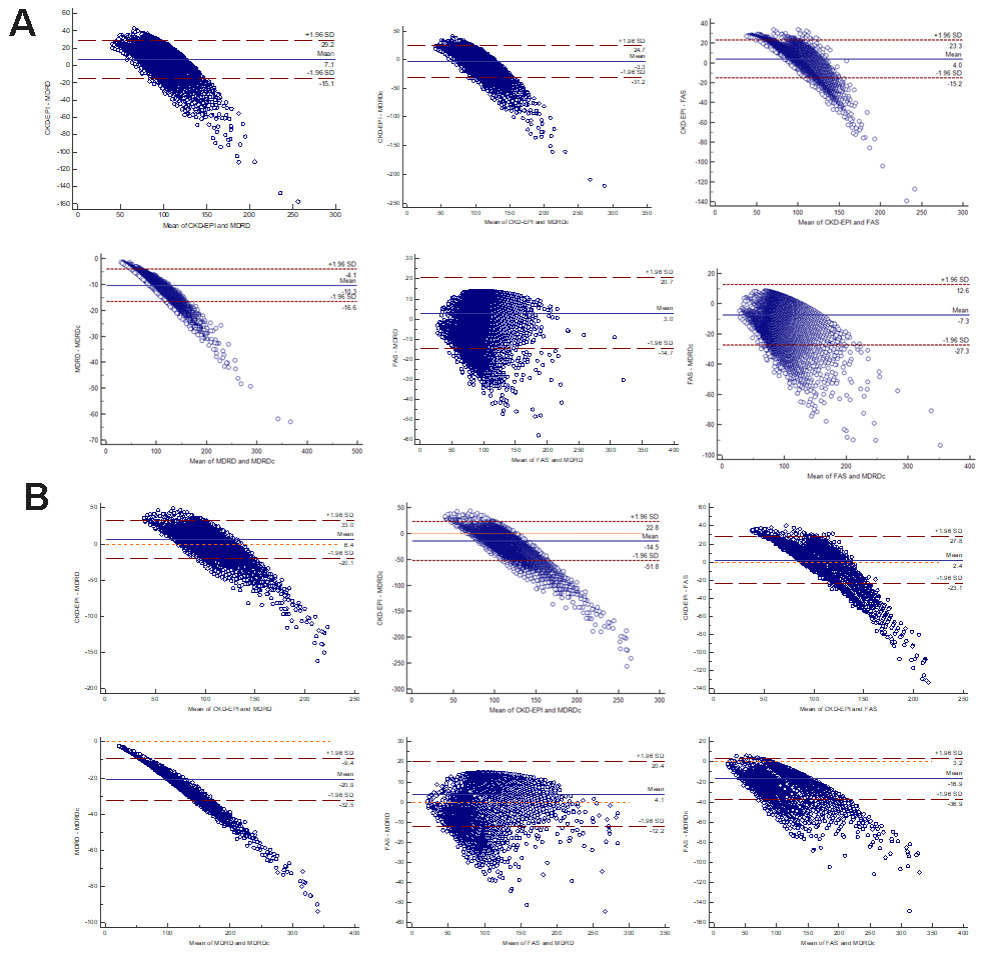
**Bland-Altman scatter plot for different equations.** (**A**) Male. (**B**) Female. CKD-EPI: Chronic Kidney Disease Epidemiology Collaboration equation; MDRD: Modification of Diet in Renal Disease Study equation; MDRDc: Chinese MDRD equation; FAS: Full Age Spectrum.

We then applied the Bland-Altman statistical method to analyze consistency between various equations. As can be seen from Figure 4, the CKD-EPI equation was consistent with the MDRD, MDRDc, and FAS equations, while the FAS equation was in good agreement with the MDRD and MDRDc equations. The MDRD and MDRDc equations showed poor consistency, and the MDRD equation was generally better than the MDRDc equation, when evaluating renal function.

### Comparative analysis of eGFR equations across different serum creatinine levels

**Figure 5:**
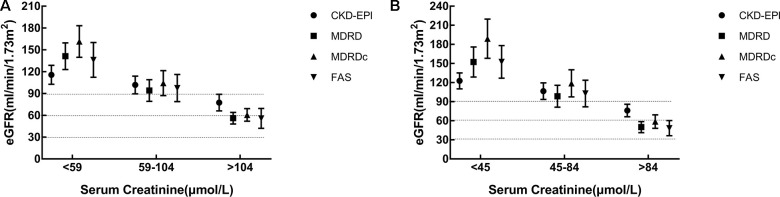
**Comparison of estimated glomerular filtration rate (eGFR) equations for different serum creatinine levels.** (**A**) Male. (**B**) Female. eGFR: evaluated glomerular filtration rate; CKD-EPI: Chronic Kidney Disease Epidemiology Collaboration equation; MDRD: Modification of Diet in Renal Disease Study equation; MDRDc: Chinese MDRD equation; FAS: Full Age Spectrum.

All subjects were divided into three groups according to our laboratory's reference range for sCr: 59-104 μmol/L for males and 45-84 μmol/L for females. It has been established that the choice of GFR equation can exert influence on CKD classification [8]. Figure 5 showed that the CKD-EPI equation yielded higher GFR values than the other equations when sCr levels exceeded the upper normal limit. Statistical differences were detected between different groups and between different equations (*P*=0.000).

### Changes in the renal function of subjects over 70 years-of-age and the application of different eGFR equations

All of the equations tested showed that eGFR decreased significantly with age in subjects over 70 years-of-age (*P*<0.05). Of the five equations tested, the MDRD and the MDRDc equations yielded higher eGFR results than the other three equations. The BIS equation yielded the lowest eGFR values (Table 3). The ICC remained consistent across all equations; ICC was 0.966 for males and 0.957 for females.

**Table 3:** A comparison of data produced by the five different estimated glomerular filtration rate (eGFR) equations in elderly subjects.

**Age**	**CKD-EPI**	**MDRD**	**MDRDc**	**FAS**	**BIS**
**Male**					
70-74	85.57±6.23	85.70±17.44	96.14±20.96	74.7±13.22	71.86±11.07
74-79	81.58±6.31	81.50±17.42	91.35±20.89	68.28±12.73	65.66±10.68
80-84	78.30±6.51	79.07±18.10	88.67±21.70	63.41±12.67	61.12±10.64
85-89	74.32±6.54	74.51±18.10	83.41±21.68	57.32±12.13	55.74±10.27
≥90	69.96±7.57	71.10±19.77	79.62±23.50	51.44±12.65	50.67±10.97
**Female**					
70-74	87.01±5.16	84.03±16.89	102.31±21.98	74.12±13.02	72.78±11.18
74-79	83.02±5.55	79.94±18.02	97.28±23.42	67.54±13.29	66.35±11.41
80-84	79.7±5.38	76.94±17.8	93.61±23.18	62.35±12.53	61.45±10.76
85-89	75.88±5.71	72.59±18.65	88.2±24.2	56.4±12.56	56.07±10.91
≥90	72.45±6.55	71.17±21.8	86.64±28.35	52.15±14.01	52.28±12.23

eGFR: estimated glomerular filtration rate; CKD-EPI: Chronic Kidney Disease Epidemiology Collaboration equation; MDRD: Modification of Diet in Renal Disease Study equation; MDRDc: Chinese MDRD equation; FAS: Full Age Spectrum; BIS: Berlin Initiative Study equation.

## DISCUSSION

In this study, we analyzed changes in sCr in a healthy population of Chinese subjects across a wide age range and then used a range of equations to determine eGFR for each subject. We found that the level and variation of sCr increased with age, especially in elder population. As reported previously, sCr increases with age in Caucasian males and females. [14]. Interestingly, we observed that these changes in eGFR varied, although the eGFR of most subjects remained unchanged with age. This finding was consistent with the results of existing studies on aging performed overseas [3, 15]. The longest-running research study of aging was the Baltimore Study (BLSA) [2], which was initiated by the National Institute of Aging (NIA) in 1958. The results arising from this earlier study showed that there was no absolute decline in renal function (sCr clearance exhibited a positive slope over time) in one third of subjects, while a proportion of participants showed a statistically significant increase in sCr clearance with age.

There are two parameters that we can use to investigate GFR: measured GFR (mGFR) and estimated GFR (eGFR). mGFR is obtained by directly measuring a certain filtration marker (such as insulin clearance, iohexol clearance rate, or the evaluation of isotope nephrograms). These evaluations are highly accurate but are complex and difficult to apply consistently in busy clinical scenarios. In contrast, eGFR is calculated by specific equations that have been established in a variety of different subject cohorts; these are convenient to use, although there is a clear need to compare the accuracy of eGFR data with mGFR data.

The first eGFR equation, the C-G equation, was established in 1976 and was based on 249 American adults aged 18 to 92-years-of-age [5]. This was followed by the development of the MDRD equation in 1999 [6]; this equation was based on 1628 patients with kidney disease and is more applicable to patients with CKD. However, there are some disadvantages associated with this method, such as the use of picric acid to determine sCr; this safety issue was addressed by the enzymatic methodology used for the corrected MDRD equation in 2006 [16]. However, this method was not applicable to Asian and elderly populations. In 2006, a Chinese version of the MDRD was published which took into account the specific characteristics of the Chinese population [17]. Although the applicability of the modified and simplified MDRD equation to determine eGFR, has been greatly improved [18], it still tends to underestimate the true value when GFR is high.

The CKD-EPI equation was first published in 2009 and was established by a cohort of 8,254 people [14]. Compared to the MDRD, this equation was validated with a population of subjects with a wider variety of clinical characteristics and a greater number of female subjects. The CKD-EPI proved to be more accurate than the MDRD [19], particularly in the elderly and when GFR>60 mL/min/1.73m^2^.

However, none of these eGFR equations specifically addressed the elderly until 2012. Although the CKD-EPI equation has been developed in adults, younger than 75 years, its use has been validated, later on, in older adults. [20]. The KDIGO guidelines subsequently recommended that the CKD-EPI equation should be used to determine the eGFR [8, 21, 22]. It was also around this time that German researchers published the BIS equation for subjects over 70 years-of-age [10]; this equation was specifically recommended for elderly patients with a GFR>30 ml/min/1.73m^2^. The BIS equation was significantly more accurate than the C-G, MDRD, or CKD-EPI equations. However, the cohorts of subjects used to validate these equations were all Caucasian. Consequently, there was a clear need to verify whether this equation was applicable to elderly subjects in China.

The most recently developed equation was the FAS equation, which was published in 2016 [9]. For the first time, this equation permitted eGFR calculations for all ages and was validated with a cohort of 735 children (2-18 years-of-age), 4,371 adults (18-70 years-of-age), and 1,764 elderly subjects (≥70 years-of-age). Results showed that for the elderly population, the FAS equation was associated with less bias, and better accuracy, than the CKD-EPI equation [23, 24].

The four eGFR equations tested in this study (CKD-EPI, MDRD, MDRDc, and FAS) showed good consistency over all age groups. In this study, we did not measure GFR directly. Consequently, it was impossible to determine which eGFR equation was the most accurate across different age groups [25]. The accuracy of the CKD-EPI equation had been previously confirmed in non-elderly patients, including Asians [26, 27], and it is recommended by the KDIGO guidelines [28]. When sCr levels exceeded the upper limit of normal, CKD-EPI equation yielded higher values for eGFR than other equations. We found that the curves of the CKD-EPI and FAS equations crossed at approximately 40 years-of-age. The curve generated by the FAS equation only began to decline beyond the age of 40 years; this is because this equation features a built-in age decline factor, 107.3/(Scr/Q), that is multiplied by 0.988^(Age - 40).^ The CKD-EPI equation assumes that renal decline has already begun by the age of 18 years; as a consequence, this equation features a factor that considers this rate of decline (0.993^Age^) and is applied from 18 years-of-age. The FAS equation has been shown to be accurate with regards to mGFR across all ages [23, 24]. Therefore, CKD-EPI equations may not be suitable for subjected with increasing levels of sCr. Our results also indicated that the MDRD and MDRDc equations are unsuitable, as they yield higher values for eGFR than the other equations [25, 29].

These findings suggested that we should be cautious when using a simple equation to stage CKD in different groups of subjects. Rather, we believe that we should choose a specific equation to determine eGFR in order to provide the most accurate and consistent information to assist clinical decision-making. The use of different eGFR equations could also lead to significant changes in the epidemiology of renal function staging [30]. The evaluation of kidney function in the elderly is highly complex. Consequently, it is very important to select a suitable eGFR equation when evaluating the elderly [31].

When estimating GFR in the elderly, the BIS equation is generally believed to be more accurate than the CKD-EPI equation [32–34]. The FAS equation was designed so that it considered the age-dependent decline in older adults, as also described by the BIS-equation. This explained why these two equations are so similar when used for the elderly. However, a previous study of elderly subjects in the community reported that the accuracy of the BIS equation was no better than that of the CKD-EPI equation [35]. Our present research showed that the BIS and FAS equations correlated well with the other three equations but yielded the lowest eGFR values in elderly subjects. Our data further suggested that the commonly used CKD-EPI equation may overestimate renal function when used to evaluate renal function in elderly Chinese cohorts. Therefore, FAS and BIS equations were better in elderly Chinese subjects.

We analyzed changes in renal function over a three-year period and found that were no significant changes in either serum creatinine or eGFR. However, each eGFR equation has a built-in age factor; consequently, we should have observed a clear decreasing trend within the 3 years. This inconsistency may have arisen due to an error in creatinine monitoring, or the influence of diet or muscle mass. It is also possible that this inconsistency was related to the fact that the age factor is incorporated into the equation in an exponential position and therefore has little influence over the final calculated results. In short, we observed that the renal function was relatively stable despite variations in age. The results of a previous meta-analysis show that mGFR remained almost unchanged prior to 40 years-of-age, and that there was a strong trend for mGFR to decline year by year after the age of 40 [23]; these previous findings differed from our current results.

There are some limitations to our research that need to be considered. First, it was impossible to determine which formula was more suitable for which age group without reference to the mGFR as the existing gold standard. Secondly, it would have been better to evaluate renal function by considering cystatin C and sCr data collectively. Thirdly, it was not possible to judge edema by clinical observation only; a better method would be to use a biochemical impedance analyzer.

In summary, our data indicate that the annual change in eGFR varied from subject to subject. There may be an age-related decline in eGFR worldwide, but there this may not be visible on a 3-year interval in this study. Furthermore, it is evident that eGFR needs to be calculated in a more accurate manner in order to facilitate accurate clinical decision-making. The eGFR equations described herein can all be used to evaluate renal function, although the results differed across different populations and sCr levels. The use of different eGFR equations may lead to significant differences when adjusting drug doses, and possibly increase the risk of serious adverse reactions. The data generated in the present study were not able to identify which specific eGFR equation should be applied for elderly subjects in China. Further research is now required to investigate the most accurate equation to use when estimating GFR in elderly Chinese persons.

## MATERIALS AND METHODS

### Subjects, blood sampling and the determination of serum creatine

We enrolled apparently healthy subjects undergoing routine medical examinations in our hospital between January 2012 and December 2014. The inclusion criteria were as follows: (1) age ≥ 18 years; (2) the results of physical examinations were all normal, including blood pressure and serological tests (liver function, blood glucose, blood lipids, tumor markers, and blood routine). Subjects were excluded if they had heart failure, edema, pleural and peritoneal effusion, severe infections, malnutrition, ketoacidosis, tumors, acute/chronic kidney disease, or any form of renal replacement therapy (hemodialysis, peritoneal dialysis, or renal transplantation); and routine urine analysis showed urinary protein levels <0.5 g/L. All participants were grouped by age into five-year categories (18-19 years, 20-24 years, 25-29 years…80-84 years, 85-89 years, and ≥90years); 70 years-of-age was considered as the threshold between non-elderly and elderly subjects.

All subjects signed contracts with the hospital. Unless special circumstances were involved, all received physical examinations in our hospital at the same time every year. Therefore, we would collect data for most subjects for three consecutive years. Our analysis used data derived from original physical examinations and sCr data derived from routine blood analysis. Serum levels of sCR were determined by an enzymatic method (Hitachi 008AS Automatic biochemical analyzer, Tokyo, Japan). Within the three-year period, the samples were all measured on the same machine using the same monitoring method. We also calculated analytical coefficients for sCr: the low-value intra-day precision was 2.91%, and the high-value intra-day precision is 0.90%. We then used five different equations to determine the eGFR of each subject.

This study was approved by the ethics committee of the Beijing Hospital (Reference: 2019BJYYEC-171-01). The ethics committee waived the requirement for informed and signed consent as the analysis only involved data arising from routine physical examination.

### Equations for the determination of eGFR

Five different equations were used to determine eGFR (Table 4). All of these equations were based on serum creatinine. The unit used to calculate eGFR in this study was mL/min/1.73m^2.^ The five published formulas use mg/dL to determine sCr. Since the biochemical analyzer available in our institution measures serum creatinine in μmol/L, our measured values for serum creatine (in mg/dL) were all divided by a pre-determined constant (88.4).

**Table 4:** The five different equations used to determine estimated glomerular filtration rate (eGFR).

**Equation**	**Name [Abbreviated name]**	**Formulae**
1	Chronic Kidney Disease Epidemiology Collaboration [CKD-EPI][5]	Male: sCr≤0.9: eGFR=141×(sCr/0.9)^-0.411^×0.993^age^ sCr>0.9: eGFR=141×(sCr/0.9)^-1.209^×0.993^age^ Female: sCr≤0.7: eGFR=141×(sCr/0.7)^-0.329^×0.993^age^ sCr>0.7: eGFR=141×(sCr/0.7)^-1.209^×0.993^age^
2	The Modification of Diet in Renal Disease Study equation [MDRD][4]	GFR=186×sCr ^-1.154^×[age]^-0.203^×0.742 (if female)
3	The Chinese version of the MDRD [MDRDc] [16]	GFR=1.75×sCr r^-1.234^×(age)^-0.179^×0.79 (if female)
4	The Full Age Spectrum equation [FAS] [7]	GFR=107.3/(sCr /Q) 2≤age≤40 years GFR=107.3/(sCr /Q)×0.988 ^(age-40)^ age>40 years
5	The Berlin Initiative Study equation [BIS][8]	GFR=3736×sCr ^-0.87^×age^-0.95^×0.82 (if female)

eGFR: estimated glomerular filtration rate; GFR: glomerular filtration rate; sCr: serum creatinine.

### Statistical methods

All data analyses were performed with IBM SPSS statistical software version 22 (SPSS Inc., Chicago, USA) and MedCalc 15 (MedCalc Software, Mariakerke, Belgium). The one-sample Kolmogorov-Smirnov test was used to test raw data for normality. Data were then compared using parametric or non-parametric tests, as appropriate. Continuous data that were normally distributed are described as means ± standard deviation, while numerical data that were not normally distributed are described as medians ± interquartile range. Comparison of eGFR changes in different age groups using chi-square test. The relationship between eGFR change and age was analyzed by single factor regression. Comparisons between different age groups were performed by analysis of variance (ANOVA) while comparisons between different equations were performed using the student’s t-test. Intraclass correlation coefficients (ICCs) and Bland-Altman scatterplots were used to evaluate the consistency of the five different eGFR equations. *P*<0.05 was considered to represent statistical significance.

## Supplementary Material

Supplementary Tables
