# Supplementary material for: The effects of aging on the renal function of a healthy population in Beijing and an evaluation of a range of estimation equations for glomerular filtration rate
Source: Aging (Albany NY). 2021 Feb 26;13(5):6904–17. doi: 10.18632/aging.202548 (PMC7993673; doi:10.18632/aging.202548)
Supplement: Supplementary Tables [file aging-13-202548-s001.pdf]

SUPPLEMENTARY TABLES

Supplementary Table 1. A comparison of serum creatinine (sCr) across different age groups in a normal male population (*P* value).

Age (years)	sCr (μmol/L)#	Age (years)														
		18-19	20-24	25-29	30-34	35-39	40-44	45-49	50-54	55-59	60-64	65-69	70-74	75-79	80-84	85-89
18-19	72.47±12.54															
20-24	76.72±10.64	0.02														
25-29	78.30±10.35	0.00	0.00													
30-34	78.48±9.91	0.00	0.00	0.45												
35-39	78.32±10.31	0.00	0.00	0.93	0.46											
40-44	78.07±10.66	0.00	0.01	0.35	0.05	0.26										
45-49	77.98±11.25	0.00	0.01	0.20	0.02	0.12	0.67									
50-54	78.00±11.86	0.00	0.01	0.22	0.02	0.15	0.73	0.95								
55-59	78.29±12.57	0.00	0.00	0.96	0.39	0.88	0.35	0.18	0.84							
60-64	77.95±12.63	0.00	0.01	0.19	0.02	0.13	0.60	0.89	0.00	0.05						
65-69	78.89±12.74	0.00	0.00	0.06	0.15	0.05	0.01	0.00	0.00	0.00	0.00					
70-74	79.92±14.38	0.00	0.00	0.00	0.00	0.00	0.00	0.00	0.00	0.00	0.00	0.00				
75-79	82.94±16.30	0.00	0.00	0.00	0.00	0.00	0.00	0.00	0.00	0.00	0.00	0.00	0.00			
80-84	84.76±18.20	0.00	0.00	0.00	0.00	0.00	0.00	0.00	0.00	0.00	0.00	0.00	0.00	0.00		
85-89	88.78±20.13	0.00	0.00	0.00	0.00	0.00	0.00	0.00	0.00	0.00	0.00	0.00	0.00	0.00	0.00	
≥90	93.74±27.83	0.00	0.00	0.00	0.00	0.00	0.00	0.00	0.84	0.05	0.00	0.00	0.00	0.00	0.00	0.00

#Data are expressed as mean ± SD.
sCr: serum creatinine.

Supplementary Table 2. A comparison of serum creatinine (sCr) levels across different age groups in a normal female population (*P* value).

Age (years)	sCr (μmol/L) #	Age (years)														
		18-19	20-24	25-29	30-34	35-39	40-44	45-49	50-54	55-59	60-64	65-69	70-74	75-79	80-84	85-89
18-19	54.61±8.83															
20-24	56.43±7.61	0.15														
25-29	56.51±7.96	0.12	0.82													
30-34	56.30±8.15	0.16	0.70	0.29												
35-39	57.16±8.11	0.05	0.03	0.00	0.00											
40-44	57.21±8.63	0.04	0.02	0.00	0.00	0.81										
45-49	58.05±8.76	0.01	0.00	0.00	0.00	0.00	0.00									
50-54	58.57±9.11	0.00	0.00	0.00	0.00	0.00	0.00	0.01								
55-59	59.57±9.87	0.00	0.00	0.00	0.00	0.00	0.00	0.00	0.00							
60-64	59.82±9.94	0.00	0.00	0.00	0.00	0.00	0.00	0.00	0.00	0.28						
65-69	60.58±11.01	0.00	0.00	0.00	0.00	0.00	0.00	0.00	0.00	0.00	0.01					
70-74	62.86±12.02	0.00	0.00	0.00	0.00	0.00	0.00	0.00	0.00	0.00	0.00	0.00				
75-79	65.57±15.03	0.00	0.00	0.00	0.00	0.00	0.00	0.00	0.00	0.00	0.00	0.00	0.00			
80-84	67.16±15.61	0.00	0.00	0.00	0.00	0.00	0.00	0.00	0.00	0.00	0.00	0.00	0.00	0.00		
85-89	70.78±18.13	0.00	0.00	0.00	0.00	0.00	0.00	0.00	0.00	0.00	0.00	0.00	0.00	0.00	0.00	
≥90	72.62±20.90	0.00	0.00	0.00	0.00	0.00	0.00	0.00	0.00	0.00	0.00	0.00	0.00	0.00	0.00	0.16

#Data are expressed as mean ±S D.
sCr: serum creatinine.

Supplementary Table 3. Changes of estimated glomerular filtration rate (eGFR) in different groups of male subjects over a 3-year period.

Age	Total number	Reduced		Unchanged		Elevated	
		N (%)	ΔeGFR[#]	N (%)	ΔeGFR[#]	N (%)	ΔeGFR[#]
18-19	547	102(18.65%)	-14.26±6.80	309(56.49%)	0.42±2.42	136(24.86%)	9.19±3.47
20-24	2775	335(12.07%)	-11.58±5.87	1624(58.52%)	0.68±2.45	816(29.41%)	8.25±3.76
25-29	3580	404(11.28%)	-9.77±4.87	2303(64.33%)	0.83±2.53	873(24.39%)	7.96±3.13
30-34	2702	266(9.84%)	-9.71±4.49	1927(71.32%)	0.70±2.48	509(18.84%)	8.22±3.54
35-39	2691	288(10.70%)	-10.41±6.02	1799(66.85%)	0.75±2.44	604(22.45%)	8.01±3.45
40-44	2822	268(9.50%)	-8.85±3.64	2069(73.32%)	0.75±2.59	485(17.19%)	8.15±3.57
45-49	2142	241(11.25%)	-9.10±4.44	1474(68.81%)	0.61±2.51	427(19.93%)	7.51±2.24
50-54	2279	274(12.02%)	-9.41±3.85	1659(72.80%)	0.65±2.51	346(15.18%)	7.69±3.00
55-59	1834	231(12.60%)	-9.28±3.82	1367(74.54%)	0.60±2.54	236(12.87%)	7.22±3.13
60-64	1376	140(10.17%)	-8.59±4.14	1086(78.92%)	0.51±2.44	150(10.90%)	6.87±1.77
65-69	1487	152(10.22%)	-8.20±3.75	1177(79.15%)	0.15±2.49	158(10.63%)	8.00±3.50
70-74	1685	162(9.61%)	-7.78±2.52	1358(80.59%)	0.16±2.58	165(9.79%)	7.27±2.23
75-79	988	109(11.03%)	-6.97±2.41	802(81.17%)	0.11±2.56	77(7.79%)	8.39±3.33
≥80	324	33(10.19%)	-7.57±2.24	266(82.10%)	0.57±1.97	25(7.72%)	9.21±4.43

[#]Data are expressed as mean ± standard deviation.
eGFR: estimated glomerular filtration rate.

Supplementary Table 4. Changes of estimated glomerular filtration rate (eGFR) in different groups of female subjects over a 3-year period.

Age	Total number	Reduced		Unchanged		Elevated	
		N (%)	ΔeGFR[#]	N (%)	ΔeGFR[#]	N (%)	ΔeGFR[#]
18-19	884	177(20.02%)	9.88±5.50	530(59.95%)	0.14±2.72	177(20.02%)	9.10±3.87
20-24	2660	483(18.98%)	9.50±5.85	1672(62.86	-0.27±2.63	505(18.98%)	8.64±3.71
25-29	2655	482(18.98%)	10.48±5.30	1669(62.86%)	0.21±2.58	504(18.98%)	8.47±4.13
30-34	1834	310(18.70%)	10.16±5.11	1181(64.39%)	0.31±2.60	343(18.70%)	8.78±4.69
35-39	2101	334(13.85%)	9.47±4.33	1476(70.25%)	0.20±2.51	291(13.85%)	7.78±3.35
40-44	2057	307(13.37%)	10.36±5.50	1475(71.71%)	0.28±2.55	275(13.37%)	7.35±2.68
45-49	1803	210(11.98%)	9.43±3.96	1377(76.37%)	0.57±2.47	216(11.98%)	7.22±1.86
50-54	1815	189(13.44%)	9.36±4.34	1382(76.14%)	0.63±2.42	244(13.44%)	7.12±2.49
55-59	1236	155(13.43%)	9.69±4.48	915(74.03%)	0.37±2.37	166(13.43%)	7.59±2.93
60-64	635	77(8.19%)	7.93±2.68	506(79.69%)	0.39±2.52	52(8.19%)	7.97±2.50
65-69	571	55(7.71%)	8.32±2.81	472(82.66%)	0.10±2.53	44(7.71%)	6.54±1.64
70-74	579	32(10.02%)	6.90±2.07	489(84.46%)	0.45±2.42	58(10.02%)	6.69±1.64
75-79	445	32(4.72%)	7.07±1.75	390(87.64%)	0.29±2.40	21(4.72%)	7.63±1.73
≥80	175	12(6.86%)	8.15±2.46	151(86.29%)	0.41±2.38	12(6.86%)	5.82±0.53

[#]Data are expressed as mean ± standard deviation.
eGFR: estimated glomerular filtration rate.

Supplementary Table 5. Data produced by the four different estimated glomerular filtration rate (eGFR) equations in all subjects.

Age (years)	Male				Female			
	CKD-EPI	MDRD	MDRDc	FAS	CKD-EPI	MDRD	MDRDc	FAS
18-19	129.79±9.97	126.71±28.85	138.72±34.00	117.33±23.73	132.52±8.42	130.44±33.39	155.62±43.25	125.21±27.18
20-24	122.50±7.42	111.88±19.25	122.33±22.54	113.51±16.61	126.69±5.90	118.00±19.79	140.79±25.35	119.92±17.23
25-29	117.74±6.53	105.15±16.55	115.22±19.46	110.95±15.01	123.13±5.87	114.19±19.42	136.77±24.97	119.89±17.56
30-34	113.76±6.05	101.37±15.25	111.46±17.98	110.55±14.34	119.27±6.08	111.25±20.51	133.86±26.54	120.56±18.95
35-39	110.06±6.16	98.90±15.61	109.15±18.47	110.93±15.07	114.61±5.57	105.94±18.15	127.72±23.48	118.54±17.44
40-44	106.38±6.20	96.88±16.21	107.31±19.29	108.81±15.72	110.52±5.77	103.46±19.71	125.17±25.68	115.92±18.96
45-49	102.75±6.23	94.98±16.27	105.53±19.38	102.63±15.26	106.20±5.55	99.42±18.73	120.47±24.43	107.55±17.46
50-54	99.54±6.28	93.41±16.71	104.04±19.97	97.28±15.07	102.55±5.50	96.73±18.76	117.41±24.49	100.98±16.88
55-59	95.78±6.29	91.38±16.91	102.02±20.25	91.03±14.63	98.16±5.30	93.02±17.53	113.04±22.83	93.02±15.23
60-64	92.81±6.13	90.43±16.99	101.20±20.40	86.37±14.02	94.94±5.16	91.20±17.48	111.00±22.85	87.70±14.56
65-69	89.01±5.95	87.73±16.42	98.27±19.71	80.12±13.06	91.20±5.19	88.73±17.61	108.14±23.00	81.54±14.04
70-74	85.57±6.23	85.70±17.44	96.13±20.96	74.78±13.22	87.00±5.15	84.02±16.88	102.30±21.98	74.17±13.02
75-79	81.58±6.31	81.50±17.42	91.35±20.89	68.28±12.73	83.02±5.55	79.93±18.02	97.27±23.42	67.54±13.29
80-84	78.30±6.51	79.07±18.10	88.67±21.70	63.41±12.67	79.69±5.38	76.94±17.80	93.60±23.18	62.35±12.53
85-89	74.32±6.54	74.51±18.10	83.41±21.68	57.32±12.13	75.87±5.71	72.59±18.65	88.19±24.19	56.40±12.56
≥90	69.96±7.57	71.10±19.77	79.62±23.50	51.44±12.65	72.45±6.55	71.16±21.80	86.64±28.34	52.14±14.01

eGFR: estimated glomerular filtration rate; CKD-EPI: Chronic Kidney Disease Epidemiology Collaboration equation; MDRD: Modification of Diet in Renal Disease Study equation; MDRDc: Chinese MDRD equation; FAS: Full Age Spectrum; BIS: Berlin Initiative Study equation.
